# Implicit Binding of Facial Features During Change Blindness

**DOI:** 10.1371/journal.pone.0087682

**Published:** 2014-01-30

**Authors:** Pessi Lyyra, Hanna Mäkelä, Jari K. Hietanen, Piia Astikainen

**Affiliations:** 1 Department of Psychology, University of Jyväskylä, Jyväskylä, Finland; 2 Human Information Processing Laboratory, School of Social Sciences and Humanities, University of Tampere, Tampere, Finland; University of Münster, Germany

## Abstract

Change blindness refers to the inability to detect visual changes if introduced together with an eye-movement, blink, flash of light, or with distracting stimuli. Evidence of implicit detection of changed visual features during change blindness has been reported in a number of studies using both behavioral and neurophysiological measurements. However, it is not known whether implicit detection occurs only at the level of single features or whether complex organizations of features can be implicitly detected as well. We tested this in adult humans using intact and scrambled versions of schematic faces as stimuli in a change blindness paradigm while recording event-related potentials (ERPs). An enlargement of the face-sensitive N170 ERP component was observed at the right temporal electrode site to changes from scrambled to intact faces, even if the participants were not consciously able to report such changes (change blindness). Similarly, the disintegration of an intact face to scrambled features resulted in attenuated N170 responses during change blindness. Other ERP deflections were modulated by changes, but unlike the N170 component, they were indifferent to the direction of the change. The bidirectional modulation of the N170 component during change blindness suggests that implicit change detection can also occur at the level of complex features in the case of facial stimuli.

## Introduction

Cognitive psychologists have discovered an astounding inability to detect considerable and obvious changes in visual scenes presented after a global transient event, for example an eye-blink or a “flicker” – a brief blank screen with a blink-like effect. Once detected, the change becomes impossible to ignore. To recover from this “change blindness” [1]–[3] – to consciously recognize the change and report on it – seems to require focal attention [4]. However, it remains controversial whether unnoticed changes are, nevertheless, registered implicitly, and if so, what kinds of representations exist outside conscious visual perception, and whether they can contribute to overcoming change blindness.

A number of studies have reported evidence of implicit representations of changes during change blindness as revealed by indirect measurement techniques in the absence of overt reportability of the change. These studies have employed behavioral [5]–[9], brain-imaging [10]–[12], and electrophysiological methods in investigation [9], [13]–[17], and have presented evidence that at least single feature changes are implicitly registered. For example, electrophysiological studies have revealed short-latency brain responses to explicitly undetected changes in complex natural scenes, with objects or their features appearing, disappearing, or changing color or location [14]. There is also evidence of implicit localization of changes as indicated both by the N2pc-component of event-related potentials (ERPs) occurring contralaterally to the changes [16], [17] and by eye-tracking studies showing the viewer’s gaze to linger in the location of the implicit changes [5]. If, as these experiments suggest, implicit representations of the changed features exist, an interesting follow-up question would be to investigate whether the implicit detection of changes occurs only at the level of single features or if it is possible to implicitly perceive changes in objects that are composed of complexes of single features.

In previous studies, the implicit registration and localization of changes involved changes in single features. It has been suggested that the detection of these types of changes does not require focal attention. According to an influential view of human perception, referred to as the feature integration theory, the distinct visual features of which coherent objects consist in human perception are correctly bound together only within the sphere of focal attention [18]–[22]. Evidence for this has been provided in visual search experiments showing inefficient, serial search for feature conjunctions and efficient, parallel search for single features [18]. Thus, if successful change detection requires focal attention and, on the other hand, if complexes of the stimulus elements lose their structural composition [23] and become randomly conjoined [19], [20] when presented outside of focal attention the complex organization of the elements in a changing stimulus cannot prima facie facilitate change detection in change blindness.

In contrast to feature integration in object perception in the visual domain, which operates by decomposing objects first into elementary parts and their edge and contour features [24], face perception has been described to operate in a holistic manner already at the first stages of visual processing [25]. Indeed, recent cognitive neuroscience research has strongly indicated that faces are perceived holistically and that basic facial features are already bound together by the brain’s subcortical face-processing route, which is involved in coarse and fast face detection [26]. Also, according to the feature integration theory, during the initial feedforward pass, visual processing of single features activates a number of potential, internally consistent feature conjunctions, the forming of which can be constrained by expectation, semantic knowledge [21], and cortical specialization [27]. As humans are specialized in perceiving faces, it is possible that changes in a facial configuration could be represented without awareness of those changes and they could also facilitate the detection of these changes. Moreover, it has been suggested that a lack of awareness, as in change blindness, does not necessarily imply lack of attention [28], and some attentional operations may support initial feature binding. For example, attention distributed over multiple objects has been shown to enable more detailed processing compared to processing of an object outside of attention, but not as much as with focused attention [21].

It has also been shown that cortical brain areas related to face perception are activated in response to facial stimuli, even without any conscious awareness of them (fusiform face area, superior temporal sulcus) [29], [30]. The fusiform face area is said to play a role in the encoding of invariant facial features, important for facial identity recognition, whereas the superior temporal sulcus is involved in processing more dynamic information such as facial expressions [31]. N170 is a component of ERPs that has been thought to reflect the representation of “the concept of a face” (structural encoding of holistic face configuration) [26]. It has been shown that realistic and schematic pictures of faces generally elicit similar N170 responses [32], [33]. The N170 response may also be sensitive to facial emotional expressions [34]–[36], although some earlier studies do not show this effect [37], [38]. In a study using an inter-ocular suppression paradigm, intact faces presented to participants below the threshold of awareness elicited an enlarged N170 response compared to scrambled faces in postero-temporal areas [30]. In another study comparing subconsciously presented emotional expressions to neutral ones, the former elicited an enhanced EPN-like response, a response sensitive to emotionally and motivationally salient stimuli, approximately 220 ms after stimulus onset [39]. These results among others on non-conscious face-perception [26], [40]–[42] indicate that facial features are bound together and that these feature complexes can be detected by the brain, even without awareness of them.

In studies measuring change detection performance, it has been shown that socially relevant changes, including changes involving faces, are often detected more easily than socially neutral ones (gradual changes in facial expressions vs. gradual color changes [43]; people vs. objects [44]; heads vs. objects: [45]). These authors have explained the more efficient change detection in socially relevant stimuli as a result of the interplay between salience and attentional effects. Compared to neutral stimuli, socially relevant stimuli draw attention for longer periods of time. Thus the earlier detection of changes in faces than in other objects may be due to the stronger allocation of attention to faces. However, even if attention plays an important role here, it is still possible that the eventual change detection depends on the perception of simple features or luminance changes in facial stimuli rather than combinations of single features. Whether complex facial configurations could be perceived implicitly and whether this could have a bottom-up effect on explicit change detection were questions left open by these studies. We reasoned that, by using controlled facial stimuli and measuring face-related ERP components, especially the N170 response, we could approach the issue of whether visual feature complexes are implicitly represented in the case of facial stimuli during the change blindness.

Using schematic faces and scattered groups of physically identical features (scrambled faces) as stimuli, we investigated the implicit detection of changes in facial and non-facial stimuli in the change blindness paradigm while recording ERPs. Four stimuli, two faces and two scrambled faces were presented at a time. Occasionally, one of the faces changed to a scrambled face or vice versa (between-category change). Alternatively, a face or scrambled face changed to another exemplar of the same category (within-category change). For faces, the within-category change of facial feature arrangement led to a change in facial expression. At the behavioral level, our main hypothesis concerned the between-category changes: we expected that changes involving the presence or absence of facial configural information in a stimulus (between-category changes) would be detected faster than changes in within-category changes. If, as expected by the social bias of attention hypothesis, more attention is allocated to intact faces than to scrambled ones, the deformation of faces should be detected faster than the formation of a face from scrambled features. For within-category changes, we expected that changes in intact faces would be more easily detected than those in scrambled faces. At the electrophysiological level, we expected to observe a modulation of the face-sensitive N170 response during change blindness, indicating implicit change detection of facial configuration. We hypothesized that an enlargement of the N170 response would be observed for the changes from scrambled features to faces, and an attenuation of the N170 response for changes in the other direction. Since previous studies have evidenced N170 response sensitivity to facial expressions [34]–[36], it was possible that the N170 response would also show an amplitude modulation for within-category changes involving faces. Because the experimental paradigm involved the presentation of repeated, unchanged visual displays interspersed by changed ones, we also expected to observe a visual mismatch negativity (vMMN) response to the changed stimuli. It has been shown that the vMMN response is elicited by regularity violations, also when participants are unaware of changes in stimuli [46]–[48].

In sum, we sought out evidence of implicit change detection in complex facial configurations during change blindness, evidence that was provided by revealing modulation of the face-sensitive N170 ERP response to configural stimulus changes of facial stimuli without explicit behavioral change detection.

## Methods

### Participants

Twenty-one healthy volunteers (fourteen females, age range 19–39 years, mean age 25.8 years) took part in the study. One participant was left-handed, the rest were right handed, and all had normal or corrected-to-normal vision. Because of timing problems with the stimulus presentation, the electroencephalogram (EEG) data on four participants were discarded. Data on seventeen participants were analyzed (eleven females, age range 19–39 years, mean age 25.7 years, all right-handed).

### Ethics Statement

According to Finnish regulations (Act on Medical Research and Decree on Medical Research 1999, amended 2010), specific ethics approval was not necessary for this study. Written informed consent was obtained from the participants before the experimental treatment. The study conforms to The Code of Ethics of the World Medical Association (Declaration of Helsinki).

### Stimuli and Procedure

The participants viewed the stimuli on a 17″ monitor (Eizo Flexscan CRT display, refresh rate 85 Hz) at a distance of 60 cm.

**Figure 1:**
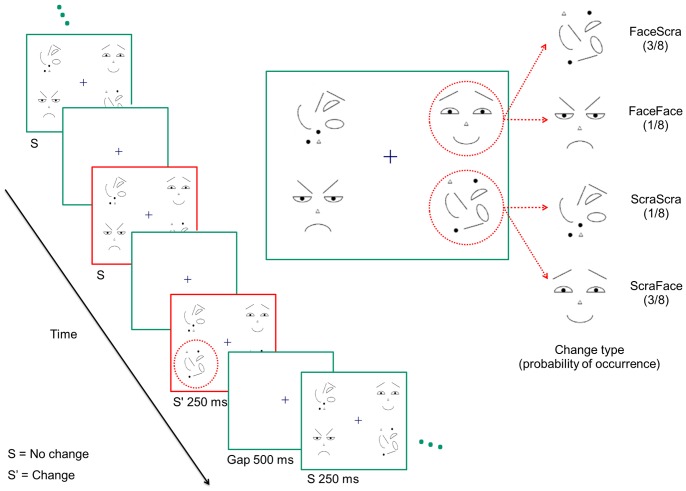
An excerpt from a stimulus sequence. The No change and Change conditions consist of an image pair separated by the blank interval within the same stimulus trial, indicated by the red frames (previous no change image+change image). ERPs were extracted from responses to these images and the preceding blank interval.

Two types of images were used: a set of three schematic faces (neutral face, happy face, and fearful face), and a set of three scattered constellations of the same facial features (scrambled faces, see Fig. 1). Randomly changing constellations of scrambled face elements were not used, since it has been shown that non-similarity of the stimuli affects the N170 response [33], [49] and, therefore, had the potential to act as a confounding factor. The scrambled and schematic faces covered roughly a similar spatial area. Four stimuli, two faces and two scrambled faces were presented at a time at four locations around a fixation cross (see Fig. 1).

Stimuli were continuously presented as flickering stimulus sequences of one to five change trials. In one trial, an original, repeatedly presented stimulus was visible for 250 ms, followed by a 500-ms non-stimulated interval (flicker), after which either the original or a changed version of it was presented for 250 ms. The first change occurred after presentation of at least three successive non-changing stimuli. Between the change trials, there were between three and seven presentations of non-changing stimuli. Thus, we used an oddball version of the flicker paradigm in which changes occurred infrequently with a probability of 20% [14], [15]. Compared to the more commonly used alternating flicker paradigm, the infrequent presentation of the changes increases their novelty and change value, which is required for the elicitation of related ERP components such as vMMN.

The changes consisted of an occasional change in one of the faces/scrambled faces. There were four different types of changes (Fig. 1). In the so-called between-category changes, an intact face changed to a scrambled face (FaceScra) and a scrambled face changed to an intact face (ScraFace). The two other types of changes were within-category changes. In a case of a face changing to another face, a change in the arrangement of local features led to a change in facial expression. In a case of scrambled faces, one scrambled face changed to another scrambled face (see Fig. 1). No two similar intact or scrambled faces were presented simultaneously. One type of change was presented at one location throughout a stimulus sequence, and the change types and locations were randomized within the experiment. A stimulus sequence consisted of twenty-seven stimulus presentations lasting 21 seconds at most. The duration of the whole experiment ranged from 42 to 60 minutes, consisting of between 143 and 205 (mean 201.0) stimulus sequences. During pilot testing with a different subject group, we tested the behavioral change detection of the different change types. We found that between-category changes were clearly more easily detected than within-category changes. Therefore, to obtain roughly an equal amount of data for each change type from the change blindness periods, we increased the number of stimulus sequences containing between-category changes; first to P = .333, and, after six participants, to P = .375 for both between-category change types. For within-category changes, we used P = .166 and P = .125 for faces and scrambled faces, respectively.

The participants were instructed to search for an infrequent change in the images and to report the change by pressing one of two adjacent buttons, depending on which side of the display they perceived the change happening. In some change detection studies, participants are required to report the change once they identify it. However, in these cases it is possible that people are somehow aware of the changes before they decide to report them, and therefore it is not clear whether the results reflect change blindness or awareness of the changes. Therefore, we used a strict criterion of change detection and instructed the participants to press the button once they could “sense” the location of the change, though no conscious recognition of the change was required [50], [51]. When the participant reported localizing the change by pressing the correct button, the stimulus sequence came to halt [52] and the participant initiated the next stimulus sequence with another button press. The change trials before the explicit report, except for the last one immediately preceding the report, made up the change blindness condition.

The experiment was divided into two blocks, each comprising one half of the experiment. In one block, the participants fixated a cross in the middle of the scene and tried to detect changes in the stimuli around the cross without changing fixation [10], [53], [54]. In the other block, participants were allowed to search freely for the change, but, to provide a contrast for the fixation condition, were instructed to look at only one stimulus at a time in the matrix. As both search strategies are frequently used in change blindness studies, and as the data analysis showed that the search strategy did not have a significant interaction effect with any of the manipulated factors, we averaged the data of the two search conditions in order to increase the power of the experiment.

### EEG-recordings and Data-analysis

EEG was recorded with Ag-AgCl electrodes from twenty-one channels (FP1, FPz, FP2, F3, Fz, F4, F7, F8, C3, Cz, C4, P3, Pz, P4, T3, T4, T5, T6, O1, O2, Oz) according to the international 10–20 system. Each channel was referred to the average of the other electrodes (common reference), amplified 10,000 times, online band-pass filtered (0.1–100 Hz, 24 dB per octave), and digitized at a 1000-Hz sampling rate. Horizontal and vertical eye movement potential was recorded bipolarly using electrodes placed laterally 1 cm from the outer canthus of left eye and 1 cm above the right eye. The impedances of all electrodes were kept below 3 kΩ. The data were further processed using Brain Vision Analyzer 2.0 (Brain Products GmbH, Munich, Germany). Channels with excessive muscular activity were omitted from the analyses. The data were offline band-pass filtered (0.1–30 Hz, 24 dB per octave) and corrected for ocular movements with the algorithm implemented in the Vision Analyzer software [55]. As we were interested in implicit change detection, we analyzed only the data concerning change blindness, that is, from the period before explicit change detection. The button press marked explicit detection, and the responses to changes immediately preceding change detection were discarded from the analyses. Epochs from 150 prestimulus to 300 ms postimulus for each stimulus condition were selected for ERP extraction. ERPs were averaged and corrected against a 150-ms pre-stimulus baseline. Data were segmented separately for the stimuli containing changes (S’ in Fig. 1) and the stimuli immediately preceding the changed pictures (S in Fig. 1). Thus, in the analyses, there were an equal number of responses to change and no-change images. Sweeps containing artifacts (maximum voltage 200 µV, minimum voltage −200 µV, maximum allowed voltage step 50 µV/ms, and maximum difference of values within the sweep exceeding 100 µV in any electrode) were discarded. The mean number of artifact-free trials in the analysis was 46.7 for ScraFace, 77.8 for ScraScra, 48.6 for FaceFace, and 51.8 for FaceScra. The number for ScraScra trials was therefore significantly greater than for others (p<.01).

Based on previous research and a visual inspection of the waveforms of grand-average ERPs, mean amplitude values were calculated for each participant with regard to three components: the P1 (90–110 ms post-stimulus), the N170 (150–170 ms post-stimulus), and vMMN (250–300 ms post-stimulus). To analyze the effects of changes in different change types, we calculated mean difference amplitudes (Change – No change) for all three components. Since the P1 and vMMN responses were distributed across the occipito-temporal channels, differential change processing between change type conditions was analyzed with an analysis of variance (ANOVA) for repeated measures. For this, we used the mean difference amplitudes from channels T5, T6, O1, and O2 as a dependent variable with Hemisphere (Left, Right), Channel (Temporal, Occipital), and Change type (Schematic to Schematic, Schematic to Scrambled, Scrambled to Scrambled, Scrambled to Schematic) as factors. The N170 amplitude analyses were based on ERPs recorded from electrodes T5 and T6, as these recording sites are typically the most sensitive to facial stimuli [32]. For N170, we performed an ANOVA analysis on the mean difference amplitudes using the factors of Hemisphere and Change type. To test whether change had an effect on ERP responses in the different change types, the difference amplitudes of all the components were also analyzed with one-sample t-tests against zero. The behavioral data were measured as the mean number of change occurrences required for explicit change detection in each Change type condition, and subjected to an ANOVA for repeated measures with the factor of Change. Bonferroni corrections were used when appropriate. An alpha level of.05 was used in all the analyses.

## Results

### Behavioral Results

Change detection performance was measured as the mean number of change occurrences required for the change to be explicitly detected within each change type condition. The results are presented in Table 1. An ANOVA showed that the detection of changes differed between change types, *F*(3, 48) = 204.4, *p*<.001. Detection was more efficient for both between-category changes compared to both within-category changes, all *p*s <.01 (Bonferroni corrected). For between-category changes, there was no difference in detection when a face changed to a scrambled face or when the opposite change occurred. However, for within-category changes, changes involving faces (i.e. the expression change) were detected more efficiently than those involving scrambled faces, *t*(16) = 5.4, *p*<.01.

**Table 1:** Mean number of change presentations required for explicit change detection.

	ScraFace	FaceScra	ScraScra	FaceFace
**N**	**1.80**	**1.84**	**3.65**	**2.67**
S.E.	.45	.29	.93	.33

### ERP Results

#### P1 component

A Channel * Hemisphere * Change type ANOVA conducted for P1 difference amplitudes (Change – No change) revealed no significant main effects or interactions between any of the factors. A further analysis (data averaged across all conditions) with a one-sample t-test against zero showed that the P1 response was not modulated by the change occurrence (*p*>.60).

#### N170 component

For the N170 difference amplitudes, a Hemisphere * Change type ANOVA revealed a main effect of Change type, *F*(3, 48) = 3.1. *p*<.05, and an interaction of Hemisphere * Change type, *F*(3, 48) = 2.5, *p*<.075. Because of this interaction, we analyzed the N170 difference amplitudes separately for electrode sites T5 and T6. The mean amplitudes of the N170 component at electrode sites T5 and T6 in all change type conditions are given in Table 2.

**Table 2:** The mean amplitudes of the N170 component in the change blindness condition.

T5				
	Between-category changes		Within-category changes	
	ScraFace	FaceScra	ScraScra	FaceFace
**µV**	−**2.92**	−**2.44**	−**2.39**	−**2.65**
S.E.	1.32	1.35	1.18	1.03
	No change	No change	No change	No change
**µV**	−**2.84**	−**2.84**	−**2.30**	−**2.30**
S.E.	1.13	1.43	1.18	1.41
	Difference	Difference	Difference	Difference
**µV**	**-.08**	**.40**	**-.09**	**-.34**
S.E.	.96	1.25	.62	1.23
**T6**				
	**Between-category changes**		**Within-category changes**	
	**ScraFace**	**FaceScra**	**ScraScra**	**FaceFace**
**µV**	−**4.34**	−**2.47**	−**3.39**	−**3.38**
S.E.	2.20	2.27	1.71	2.00
	No change	No change	No change	No change
**µV**	−**3.84**	−**3.25**	−**3.05**	−**3.42**
S.E.	2.73	2.51	1.59	2.11
	Difference	Difference	Difference	Difference
**µV**	**-.60**	**.78**	**-.34**	**-.04**
S.E.	1.14	1.50	1.17	1.08

**Figure 2:**
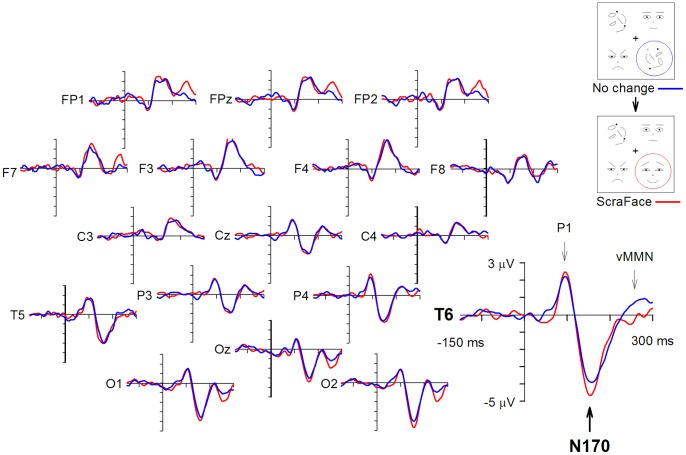
Grand average ERPs during change blindness in the FaceScra condition. The No change image was preceded by an identical image, and the Change image by the No change image. The timelines start from the onset of the Change or No change image after the blank interval.

**Figure 3:**
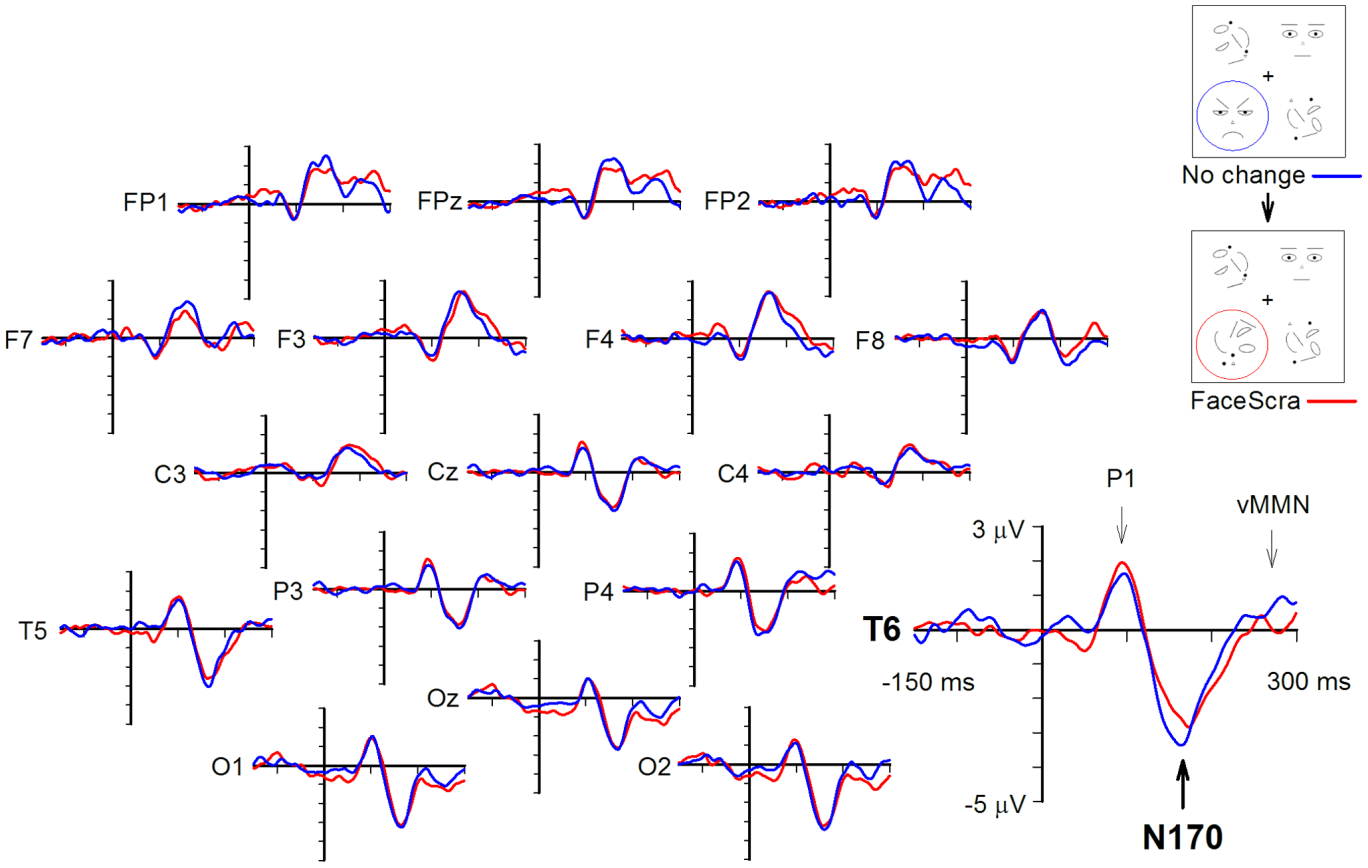
Grand average ERPs during change blindness in the ScraFace condition. The No change image was preceded by an identical image, and the Change image by the No change image. The timelines start from the onset of the Change or No change image after the blank interval.

A one-way ANOVA conducted for the data from electrode site T5 showed no significant main effect of Change type. For electrode T6, an ANOVA revealed a significant main effect of Change type, *F*(3, 48) = 3.7, *p*<.02. Pairwise comparisons revealed significant differences between FaceScra and ScraFace, and also between FaceScra and ScraScra conditions, *t*(16) = 2.6, p<.05 for both. Finally, we checked, using one-sample t-tests against zero, whether there was a significant modulation of the N170 response at T6 electrode by a change in different change type conditions. These analyses revealed significant differences in N170 responses to changed vs. unchanged stimuli in both between-category change conditions: an enhancement of the N170 amplitude by scrambled-to-face changes (ScraFace), *t*(16) = 2.2, *p*<.05, and an attenuation of it by face-to-scrambled changes (FaceScra), *t*(16) = −2.1, *p*<.05. Neither within-category changes resulted in a significant N170 amplitude modulation. The N170 responses to unchanged and changed stimuli in both between-category change conditions are illustrated in Figures 2 and 3. The mean amplitudes of the N170 component at electrode sites T5 and T6 in all change type conditions are given in Table 2.

#### vMMN component

A Hemisphere * Channel * Change type ANOVA on the vMMN responses revealed no main effect or interaction involving Change type. The main effect of Hemisphere was significant, *F*(1,16) = 4.7, *p*<.05, indicating that the overall response modulation by different change types was greater in the right than left hemisphere. Further one-sample t-tests revealed that the vMMN response was modulated by the change occurrence in the right hemisphere, *t*(16) = −2.5, *p*<.05, but not in the left hemisphere, *t*(16) = 1.4, *p* = .11. The mean amplitudes of the vMMN responses averaged over all the electrode sites and change type conditions are given in Table 3.

**Table 3:** The mean amplitudes (µV) of the vMMN response in the change blindness condition.

	Left hemisphere	Right hemisphere
**Change**	**−.39**	**−.27**
S.E.	.90	1.06
**No change**	**−.24**	**.09**
S.E.	.81	1.09
**Difference**	**−.15**	**−.37**
S.E.	.57	.43

## Discussion

We investigated the implicit detection of changes in visual stimuli containing feature complexes by presenting schematic faces and scrambled faces as stimuli in a change blindness paradigm. In addition to measuring behavioral change detection performance, we also measured event-related potentials in the change blindness period. The stimuli were presented in a matrix of two intact and two scrambled faces, with one changing in one of four possible change directions (intact to scrambled, intact to another face, scrambled to intact, or scrambled to another scrambled). The results showed that behavioral change detection was clearly more efficient for between-category changes, i.e., a scrambled face changing to a coherent face or vice versa, as compared to within-category changes, i.e., an intact face or a scrambled faces changing to another face/scrambled face, respectively. More importantly for the present study, we found that even during change blindness, changes in configurations of simple features (formation or deformation of a coherent facial image) significantly influenced the amplitude of the face-sensitive N170 response. A change from a scattered positioning of the local features (scrambled face) to a face-like configuration resulted in increased N170 amplitudes, whereas disintegration of an intact face into a scrambled one led to decreased N170 amplitudes. This result shows that the visual system implicitly, in the absence of overt reportability, processes information about the facial configuration of the changed stimuli that consist of the same elementary components. The present results show that during change blindness, the brain is capable of integrating single features into feature complexes at relatively early processing stages in the case of facial configurations. These findings are compatible with the suggestion that the N170 response reflects structural encoding of the holistic face configuration [26]. Interestingly, an earlier P1 response (90–110 ms post-stimulus) was not at all modulated by the changes in stimulus configurations.

At 250–300 ms after stimulus presentation, a sustained change-related enhanced negativity developed at the posterior electrode sites. Since the changes were presented infrequently, in a pseudo-random manner (i.e. oddball condition), this negativity is most likely visual mismatch negativity. In the same latency range, the change-related N2pc [16], [17] and attentional negativity [56] have been observed, the first also during change blindness. Further studies are needed to determine whether this negativity reflects “genuine” vMMN elicited by regularity violation [47] or the effects of spatial attention (N2pc). It is notable that this differential negative deflection, like the N170 modulation, was more pronounced in the right hemisphere, which specializes in the processing of visual configural information [57]. Furthermore, vMMN studies using facial emotional expressions as deviant stimuli have reported similar right hemispheric dominance in the vMMN response [47], [48].

Previous findings of change detection performance have shown that changes involving faces or other socially relevant stimuli are more easily detected than changes in socially neutral objects [43]–[45], [58]. Our results replicated and built on these results. In previous studies, the superior performance associated with social stimuli has been explained by attentional bias to these stimuli. This can also explain our observation that faces changing to scrambled faces or faces changing their expression were more efficiently detected than non-faces changing to other non-faces. However, our results also revealed the efficient detection of changes from scrambled faces to faces. If the bias to social stimuli results from attention allocation to social stimuli during the presentation of pre-change stimuli, the deformation of faces should have been detected more efficiently than the formation of a face from a scrambled one. However, in our data, the forming of a face from a scrambled face was detected as efficiently as the deforming of a face. Moreover, the forming of a face was detected more efficiently than a change in face expression. Thus, our data cannot be explained merely by the social bias of attention hypothesis. A plausible explanation is that the visual system is capable of implicitly detecting changes in facial structure and that these changes, then, draw focal attention to the change location before explicit detection of the change. At the neural level, the subcortical network that responds to faces and modulates subsequent cortical activity may support the implicit holistic representations and shifts of attention [26]. Thus, the N170 modulation can be seen as a marker of an ability to discern the presence and absence of facial configuration in visual stimuli, which may in turn be a pre-requisite for the attentional shift required for the change detection of facial stimuli. It may be that non-facial stimuli are not processed implicitly to the same extent.

Despite these observed findings, our results do not necessarily contest the view that focal attention is required for the changes to be detected consciously, in the sense that a voluntary behavioral report can be given on the change [4]. Even if attention could be captured in a bottom-up manner before the explicit change detection [16], [17], [52], [59], the eventual explicit change detection could nonetheless require focal attention. Change blindness studies have revealed a bias of spatial attention toward the change location, as indicated by modulations of spatial attention related ERPs (N2pc) by the change location in change blindness [16], [17], confined perhaps to the change presentation immediately preceding the one leading to eventual reported detection [16], [17], [59]–[61].

As attention is held to be dissociable from awareness [28], it is possible to explain our results concerning implicit feature binding partly in terms of the interplay between attention and awareness effects. As it has been suggested, an automatic shift of implicit spatial attention precedes focused attention and explicit change detection in change blindness. This could allow the implicit binding of facial features outside the sphere of focal awareness. According to the feature integration theory, multiple possible combinations of visual stimulus features and elements are spontaneously formed in the first feedforward pass of visual processing [21], [62], [63]. The role of focused attention, as in visual serial search, is to select the correct conjunctions and provide more detailed spatial information about its objects [21]. However, already before the attentional constraints of reentrant processing, ontogenic factors as well as cortical specialization can constrain feature combinations, as presumably in the case of face perception [20], [21]. In addition, the participants may have deployed a broader window of attention before the initial localization of the change, and narrowed it down after the change localization. With attention distributed over multiple items in the scene, this could have enhanced the processing of the items, although to a less extent for each of them as compared to when being a sole target of focused attention.

Because of the privileged status and dedicated brain mechanisms of face perception, the evidence of feature integration observed in this study can only support implicit configural processing of facial features. It is not possible to draw any further conclusions about instantaneous implicit configural processing in general. Moreover, in our study, the same three configurations were used as nonfacial stimuli throughout the experiment, and it is possible that the participants may have learned these specific constellations of elements in the progression of the experimental task. It would require change detection studies using randomly changing combinations of features as changes to address implicit visual processing of feature combinations in general.

Change blindness is primarily a failure of the conscious access required for reporting the presence of change. If, as suggested by some theorists [64], access to the contents of focal attention is limited within the ample contents of visual awareness, it could be that the participants were aware of the changing stimuli at some unreportable level. As mentioned above, explicit change detection may be preceded by a feeling of change, and participants can even wait for one presentation cycle before reporting the change, to be sure that they have detected it. The change blindness period may thus be contaminated by initial explicit change detection, especially if the change detection task is easy, as in the between-category change conditions. However, in the present study, we used a sensitive criterion for change blindness, requiring only localization of the change rather than conscious recognition of it. We also excluded the trials immediately preceding the report from the ERP data concerning the change blindness condition. Therefore, we think that our ERP results reflect implicit change detection, defined as registration in the brain of the presence of change in the display, notwithstanding the failure to explicitly report it.

It has been found in a number of studies that N170 responses to facial stimuli are reduced when preceded by the same facial stimuli, or even by different stimuli of the same category, especially compared to N170 responses to the same facial stimulus preceded by a non-facial stimulus [65]–[68]. A similar adaptation effect is evident in change blindness studies using facial and non-facial stimuli, namely if the changed facial stimuli are preceded by relatively similar facial stimuli. Thus, it is possible that the adaptation effect differs across change conditions and contributes to the differential processing of changes between the within- and between-category conditions. The lack of N170 modulation in the FaceFace situation could therefore be partly due to this adaptation-related amplitude reduction. A study by Ganis and Schendan [67] made a direct comparison to determine whether this kind of an effect is due to adaptation by previously presented faces or to an increase of amplitude caused by a non-face adaptor stimulus. It was found that only adaptor faces, not adaptor objects, affected the N170 amplitudes to the adapted faces relative to a baseline. Hence in our study, the adaptation effect may have concerned only the FaceFace condition, and not necessarily others, for example the ScraFace condition. Nevertheless, in these adaptation studies, the adaptor stimulus has been presented before the target in a conscious condition. In our study, the changed facial stimuli are implicitly presented and preceded by more than one presentation of the stimuli. The role of the preceding stimuli’s adaptation effect on the changed facial stimuli is an interesting question for future change blindness studies using facial stimuli. The probabilities of occurrence and lengths of stimulus sequences differed between the within- and between-category conditions, and this may have had some minor adaptation- or task-related effects on behavioral and ERP-results. However, the probabilities and lengths were similar within the between- and within-category change conditions, respectively. Neither the adaptation or presentation frequency issues concern the bidirectional N170 modulation observed in the between-category change conditions.

Our results not only corroborate previous theories of change blindness by showing that single visual features are represented and compared in the memory during change blindness [9], [13]–[15], but also by demonstrating that the visual representation is relatively organized in the case of facial stimuli even outside focal attention and awareness. If changes are implicitly represented, then it is an open question whether change blindness is due to a failure of memory, a failure of a comparison process between pre- and post-change representations, or the inability to access information about the changes and to report them explicitly [3]. The present results concerning the N170 component do not cast light on whether the representations of the original and modified displays were compared or not, since the amplitude of the N170 response seemed to only reflect the appearance or disappearance of a facial configuration in the display, not the processing of change in them. Instead, the vMMN response seemed to be sensitive to the changes in a more general way: a similar deflection was elicited by all types of changes, highlighting the processing of change rather than changed features in the visual display. The vMMN-modulation is thus difficult to explain without postulating some kind of a comparison process for pre- and post-change representations.

In sum, we found that behaviorally undetected changes in facial configurations during the change blindness nevertheless affected the face-sensitive N170 ERP response. The N170 modulation was elicited by both the formation and deformation of a face during change blindness, which suggests that implicit representations of complex facial stimuli can exist during change blindness. On the basis of the present study, it cannot be established whether this holds for other types of complex stimuli. Nevertheless, our results may help us to understand what kind of information is retained across interruptions of stimulation and why changes involving facial stimuli seem to be more easily detectable than non-facial ones.
